# Identification of competitive endogenous RNAs network in breast cancer

**DOI:** 10.1002/cam4.2099

**Published:** 2019-04-01

**Authors:** Xiaojin Wang, Jiahui Wan, Zhanxiang Xu, Shijun Jiang, Lin Ji, Yutian Liu, Shuwen Zhai, Rongjun Cui

**Affiliations:** ^1^ Department of Biochemistry and Molecular Biology Mudanjiang Medical University Mudanjiang China; ^2^ Department of Cardiovascular Medicine Hongqi Hospital Affiliated to Mudanjiang Medical University Mudanjiang China

**Keywords:** breast cancer, ceRNA network, GO enrichment analysis, KEGG pathway analysis, prognosis, survival analysis

## Abstract

**Background:**

MiRNAs can regulate gene expression directly or indirectly, and long noncoding RNAs as competing endogenous RNA (ceRNAs) can bind to miRNAs competitively and affect mRNA expression. The ceRNA network is still unclear in breast cancer. In this study, a ceRNA network was constructed, and new treatment and prognosis targets and biomarkers for breast cancer were explored.

**Methods:**

A total of 1 096 cancer tissues and 112 adjacent normal tissues to cancer from the TCGA database were used to screen out significant differentially expressed mRNAs (DEMs), lncRNAs (DELs), and miRNAs (DEMis) to construct a ceRNA network. Gene ontology (GO) and Kyoto Encyclopedia of Genes and Genomes (KEGG) pathway enrichment analysis were used to predict potential functions. Survival analysis was performed to predict which functions were significant for prognosis.

**Results:**

From the analysis, 2 139 DEMs, 1 059 DELs, and 84 DEMis were obtained. Targeting predictions for DEMis‐DELs and DEMis‐DEMs can yield 26 DEMs, 90 DELs, and 18 DEMis. We performed GO enrichment analysis, and the results showed that the upregulated DEMs were involved in nucleosomes, extracellular regions, and nucleosome assembly, while the downregulated DEMs were mainly involved in Z disk, muscle contraction, and structural constituents of muscle. KEGG pathway analysis was performed on all DEMs, and the pathways were enriched in retinol metabolism, steroid hormone biosynthesis, and tyrosine metabolism. Through survival analysis of the ceRNA network, we identified four DEMs, two DELs, and two DEMis that were significant for poor prognosis.

**Conclusions:**

This study suggested that constructing a ceRNA network and performing survival analysis on the network could screen out new significant treatment and prognosis targets and biomarkers.

## 1 INTRODUCTION

Breast cancer is one of the most common types of cancer worldwide and is the second leading cause of death in American women.^1^, ^2^ The prevalence of breast cancer is increasing.^3^ Mammographic density, body fat, and diet are the risk factors for breast cancer.^4^, ^5^ The 12th St Gallen International Breast Cancer Conference proposed that breast cancer be divided into four subtypes using immunohistochemical definitions of estrogen and progesterone receptors, the human epidermal growth factor receptor 2 (HER2), and the Ki‐67 labeling, index including Luminal A, Luminal B, HER2, and Triple negative (ductal) subtypes.^8^ The treatment methods include surgery, radiation therapy, endocrine therapy, chemical therapy, and bio‐targeted therapy. In recent years, targeted therapy has become the research focus. However, certain patients have high recurrence rates and drug resistance, the mechanisms of which are still unclear. Currently, approximately 60% of breast cancers are first detected through mammographic screening,^9^ but several studies have suggested that cancers diagnosed by mammographic screening may represent overdiagnosis.^10^, ^11^ It is urgent to find novel diagnostic and prognostic biomarkers and new targeted therapies for breast cancer.

The competing endogenous RNA (ceRNA) hypothesis revealed a new mechanism for interactions between RNAs.^12^ MicroRNAs play an important role in the development of tumors, the 5' regions of which can bind to sequences with partial complementarity on the target RNAs' 3'UTRs, called microRNA recognition elements, usually inhibiting the expression of the target genes.^13^, ^14^ The hypothesis supposed that a certain concentration of ceRNAs could bind to miRNAs competitively to regulate the expression of the target mRNAs. The RNA‐microRNA regulatory pathway includes microRNAs and the transcriptome (the protein coding genes, pseudogenes, and long noncoding RNAs [lncRNAs]). It has been reported that the pseudogene PTENP1 could bind to and compete for microRNAs, increasing the expression levels of PTEN.^15^ Mouse breast cancer Pbcas4 is the pseudogene of human breast cancer AS4 that might regulate the expression of breast cancer through binding mir‐185.^16^ LncRNAs are more than 200 nucleotides in length and play an important role in many biological processes.^17^ Li et al reported that SNHG7 might act as a ceRNA to regulate GALNT7 expression by inhibiting miR‐34a in CRC cell lines.^18^ In addition, it was found that NEAT1 might as a ceRNA for hsa‐miR‐377‐3p, regulating its endogenous targets E2F3 in NSCLC.^19^ There is increasing evidence that ceRNA might be important in the progression of cancer. In this study, we used the TCGA database to obtain normal and tumor samples of breast cancer, and the edgeR package was used to detect the differentially expressed mRNAs (DEMs), lncRNAs (DELs), and miRNAs (DEMis). We then constructed ceRNA networks and performed an overall survival (OS) analysis. In addition, we further predicted the potential functions and regulatory pathways by performing gene ontology (GO) and Kyoto Encyclopedia of Genes and Genomes (KEGG) pathway enrichment analysis.

## 2 MATERIALS AND METHODS

### 2.1 Gene data

The RNA sequence data of 1 208 cases of breast cancer were extracted from the TCGA database (https://portal.gdc.cancer.gov/). The 1 208 samples include 1 096 cancer tissues and 112 adjacent normal tissues to cancer. All file data were downloaded using the GDC Data Transfer Tool. Since the data come from the TCGA database, no further approval was required from the Ethics Committee. Gene expression dataset GSE96670 was downloaded from GCBI (https://www.gcbi.com.cn/gclib/html/index, version 1.4.2).

### 2.2 Data processing

The RNA and miRNA sequence data were derived from the Illumina HiSeq_RNASeq and the Illumina HiSeq_miRNASeq sequencing platforms. The RNA sequences included mRNA sequences and lncRNA sequences, and we mainly used Perl and R language to analyze and interpret the RNA data.

### 2.3 Identification of mRNAs (DEMs), lncRNAs (DELs), and miRNAs (DEMis)

We used Perl *(* http://www.perl.org/, version 5.22.3) to extract the gene matrix and used Ensembl (http://asia.ensembl.org/index.html, version 94) to transform the Ensembl numbers to gene names. We then extracted lncRNAs and mRNAs using Perl. EdgeR (http://bioinf.wehi.edu.au/edgeR/, version 3.22.5) was used to analyze the differential expression of mRNAs, lncRNAs, and miRNAs. *P* < 0.01 and |logFC| ≥ 2 were set as the cutoff criteria. The differential expression analysis of the GSE96670 dataset was performed using the GCBI website.

### 2.4 Construction of a ceRNA network

First, we predicted interactions between lncRNA and miRNAs using the miRcode database (http://www.mircode.org/index.php). Next, the miRDB, mirTarBase and Target Scan databases were used to retrieve miRNA targeting mRNAs. Finally, a ceRNA network was constructed using the Cytoscape 3.5.1 online website (http://www.cytoscape.org/).

### 2.5 GO and KEGG pathway enrichment analysis

Gene ontology enrichment analysis was performed with The Database for Annotation, Visualization and Integrated Discovery (DAVID, https://david.ncifcrf.gov/summary.jsp), and FDR < 0.05 was set as the cutoff criterion. KEGG pathway analysis was applied by an R package (http://www.bioconductor.org/packages/release/bioc/html/DOSE.html, version 3.6.1), and the threshold was *P *< 0.05. GO and KEGG pathway analysis were used to predict potential functions.

### 2.6 Survival analysis

The Survival package of R (https://CRAN.R-project.org/package=survival, version 2.42‐6) was used to perform survival analysis of DEMs, DELs and DEMis from the network, setting *P* < 0.05 as the cutoff criterion.

### 2.7 Cell lines

The MCF‐7 and BCAP‐37 human breast carcinoma cell lines were obtained from the Chinese Academy of Sciences Committee from the Type Culture Collection Cell Bank (Shanghai, China). All cell lines were cultured in DMEM (HyClone) supplemented with 10% FBS (Gibco‐BRL, Gaithersburg, MD) and were maintained in a humidified atmosphere containing 5% CO_2_ at 37°C.

### 2.8 RNA extraction and qRT‐PCR analysis

Total RNA was isolated using TRIzol reagent (Invitrogen) according to the manufacturer's instructions. Quantitative real‐time PCR (qRT‐PCR) assays were performed using SYBR Premix Ex Taq (Vazyme, Nanjing, China) and were carried out in an ABI7500 RT‐PCR System (Applied Biosystems, Foster City, CA). The primer sequences are listed in Table 6. All the experiments were performed in triplicate.

### 2.9 Cell transfection

The siRNAs of human LINC00536 were synthesized by GeneWiz (Beijing, China). The siRNA lentivirus vectors were transfected into cells using Lipofectamine 2000 for 48 hours. The final concentration of the siRNAs used was 100 nM. The siRNA sequences are shown in Table 6.

### 2.10 Cell proliferation

After transfection, cells were seeded into each well of 96‐well plates at a density of 2 000 cells per 100 μL. Cell proliferation was measured using CCK‐8 (Dojindo Laboratory, Kumamoto, Japan) following the manufacturer's protocol. The reagent was added in the media and was incubated for 24, 48, 72, and 96 hours. Aliquots were taken, and the absorbance of each well was measured at a 450‐nm wavelength.

## 3 RESULTS

### 3.1 Identification of DELs, DEMs, and DEMis

**Figure 1:**
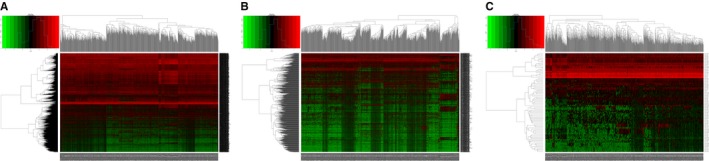
(A‐C) Heatmaps of the expression levels of differentially expressed mRNAs (DEMs), lncRNAs (DELs), and miRNAs (DEMis). The red represents upregulated expression, and the green represents downregulated expression

**Figure 2:**
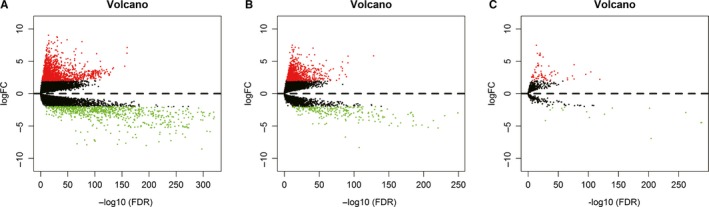
(A‐C) Volcano plots of the expression levels of differentially expressed mRNAs (DEMs), lncRNAs (DELs), and miRNAs (DEMis)

There were 1 096 cancer tissues and 112 adjacent normal tissues to cancer. EdgeR was applied to identify the differentially expressed DEMs, DELs, and DEMis (*P* < 0.01). From a total of 2 139 DEMs, 1 375 upregulated mRNAs, and 763 downregulated mRNAs (Table 1, Figures 1A and 2B), 842 upregulated DELs and 217 downregulated DELs (Table 2, Figures 1B and 2B), and 65 upregulated DEMis and 19 downregulated DEMis (Table 3, Figures 1C and 2C) were detected. The top 10 from each list are shown in the Tables. Full information for the DEMs, DELs, and DEMis is shown in Tables [Link], [Link], [Link].

**Table 1:** The top 10 upregulated and downregulated DEMs

	logFC	LogCPM	*P* value	FDR
*Up_regulated*				
COL10A1	7.118028	7.224525	3.68E‐162	2.47E‐160
MMP11	6.248933	8.219612	4.62E‐162	3.10E‐160
NEK2	4.257205	4.689387	7.18E‐158	4.56E‐156
PKMYT1	3.996334	4.276772	9.65E‐137	4.74E‐135
KIF4A	3.844023	4.776918	3.12E‐135	1.49E‐133
PCLAF	3.312735	4.260502	4.49E‐131	2.03E‐129
HSD17B6	2.999762	2.421172	1.94E‐129	8.56E‐128
SPC25	2.823913	2.880249	5.22E‐129	2.29E‐127
ASF1B	3.06672	4.749179	1.86E‐127	7.89E‐126
CDC25C	3.391866	2.594102	3.24E‐125	1.35E‐123
*Down_regulated*				
CKM	−8.33431	5.327362486	0	0
ACTA1	−7.00513	6.405634411	0	0
MYLPF	−6.96877	2.486430021	0	0
PYGM	−6.94851	3.960465036	0	0
SLN	−6.47733	1.835770736	0	0
TNNC2	−6.3626	3.038754726	0	0
ACTN3	−6.34792	0.979270699	0	0
KLHL41	−6.30849	3.167708051	0	0
ATP2A1	−6.15814	3.918391453	0	0
TNNC1	−6.15193	2.951248376	0	0

DEMs, differentially expressed mRNAs; FDR, false discovery rate.

**Table 2:** The top 10 significantly upregulated and downregulated DELs

	logFC	LogCPM	*P* value	FDR
*Up_regulated*				
LINC01614	5.845128953	9.225231521	2.82E‐131	6.63E‐129
LINC01705	5.797680513	6.174313069	3.20E‐94	3.28E‐92
LINC00922	4.773382112	6.863968286	5.47E‐94	5.53E‐92
LEF1‐AS1	2.605666782	5.532237315	6.26E‐92	5.92E‐90
C6orf99	2.96724319	7.130640435	2.69E‐90	2.30E‐88
LINC02544	3.910602341	8.19158572	5.76E‐86	4.47E‐84
LINC01561	4.917189042	5.594160253	6.25E‐86	4.81E‐84
AC134312.5	3.528028606	7.018888096	6.61E‐86	5.05E‐84
AC011893.1	4.152400037	4.687553409	4.34E‐84	3.17E‐82
AL391845.2	2.884543944	4.826658418	7.81E‐82	5.44E‐80
*Down_regulated*				
AP001528.2	−2.999793761	7.450241343	5.09E‐254	4.43E‐250
LINC02202	−2.969706049	6.66995045	2.00E‐253	8.70E‐250
LINC01537	−3.405075658	5.771695724	1.24E‐234	3.61E‐231
TRHDE‐AS1	−5.306126275	8.294236076	9.44E‐225	2.05E‐221
AL031316.1	−4.180005438	6.420712498	5.20E‐218	9.04E‐215
AC087482.1	−5.194294126	4.659880012	2.15E‐210	3.12E‐207
AL356218.1	−4.851972598	3.731532256	1.48E‐201	1.84E‐198
ALDH1L1‐AS2	−4.840960434	5.154938076	7.41E‐193	8.06E‐190
LINC01697	−4.213381929	6.264177904	4.92E‐189	4.76E‐186
AL445426.1	−3.537422352	4.436083312	6.91E‐189	6.01E‐186

DELs, differentially expressed lncRNAs; FDR, false discovery rate.

**Table 3:** The top 10 significantly upregulated and downregulated DEMis

	logFC	LogCPM	*P* value	FDR
*Up_regulated*				
hsa‐mir‐21	2.208979502	17.92834134	1.52E‐121	7.24E‐120
Hsa‐mir‐96	3.357530464	5.371537344	2.14E‐106	8.16E‐105
hsa‐mir‐183	2.981410798	14.11987777	5.15E‐98	1.63E‐96
hsa‐mir‐141	2.267084471	10.90341625	2.33E‐78	5.54E‐77
hsa‐mir‐592	4.484193593	2.350198078	2.43E‐78	5.54E‐77
hsa‐mir‐429	2.743466898	7.008958443	7.46E‐76	1.64E‐74
hsa‐mir‐200a	2.141789311	9.91017151	1.47E‐67	3.00E‐66
hsa‐mir‐182	2.398120181	15.5717091	2.37E‐67	4.67E‐66
hsa‐mir‐210	3.192893719	9.387587708	4.83E‐51	6.56E‐50
hsa‐mir‐7705	2.936658334	0.756073613	2.48E‐47	3.08E‐46
*Down_regulated*				
hsa‐mir‐133a‐1	−6.781539585	4.767162065	0	0
hsa‐mir‐133a‐2	−6.736299097	4.572519553	0	0
hsa‐mir‐1‐2	−5.971661466	5.195357754	0	0
hsa‐mir‐1‐1	−5.957465408	5.103347949	0	0
hsa‐mir‐486‐1	−4.485388317	6.636918674	6.73E‐291	7.68E‐289
hsa‐mir‐486‐2	−4.483448027	6.62762192	1.61E‐289	1.53E‐287
hsa‐mir‐139	−2.923595546	6.342438722	2.38E‐264	1.94E‐262
hsa‐mir‐133b	−6.94249258	3.437648536	1.23E‐206	8.76E‐205
hsa‐mir‐145	−2.246667335	10.85191627	5.51E‐204	3.50E‐202
hsa‐mir‐378a	−2.231795003	8.59715757	2.39E‐142	1.36E‐140

DEMis, differentially expressed miRNAs; FDR, false discovery rate.

### 3.2 GO and KEGG pathway enrichment analysis

**Figure 3:**
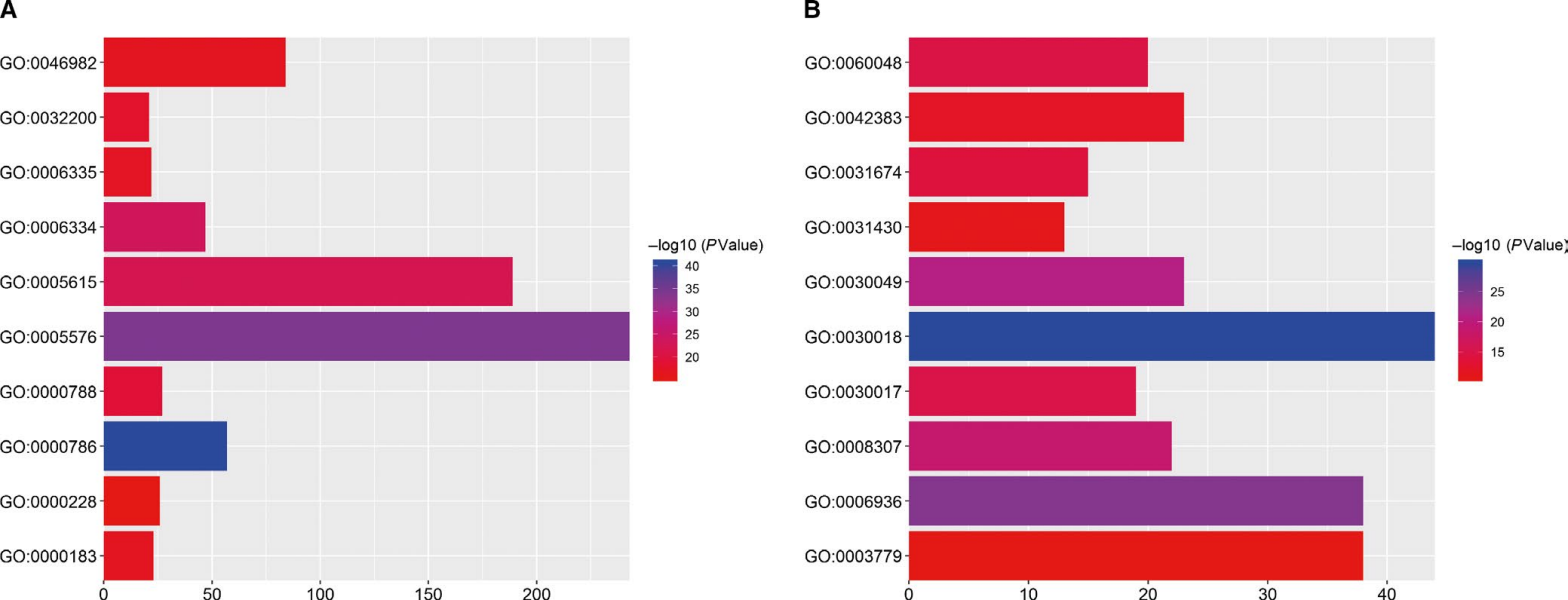
Top 10 pathways identified in the gene ontology (GO) enrichment analysis in differentially expressed mRNAs (DEMs). (A) Upregulated and (B) downregulated DEMs

To better understand the potential functions of the identified genes, we performed GO and KEGG pathway enrichment analysis. We imported the up‐ and downregulated DEMs to the DAVID and GO analysis platforms and then selected the top 10 most enriched GO terms. Four biological processes, five cellular components, and one molecular function were identified for the upregulated DEMs (Figure 3A), and three biological processes, five cellular components, and two molecular functions were identified for the downregulated DEMs (Figure 3B). For upregulated DEMs, the biological processes included nucleosome assembly, telomere organization, chromatin silencing at rDNA and DNA replication‐dependent nucleosome assembly; the cellular components included nucleosome, extracellular region, extracellular space, nuclear nucleosome, and nuclear chromosome; and the molecular function included protein heterodimerization activity. For the downregulated DEMs, the biological processes included muscle contraction, muscle filament sliding, and cardiac muscle contraction; the cellular component included Z disk, sarcomere, I band, sarcolemma, and M band; and the molecular function included structural constituents of muscle and actin binding.

**Figure 4:**
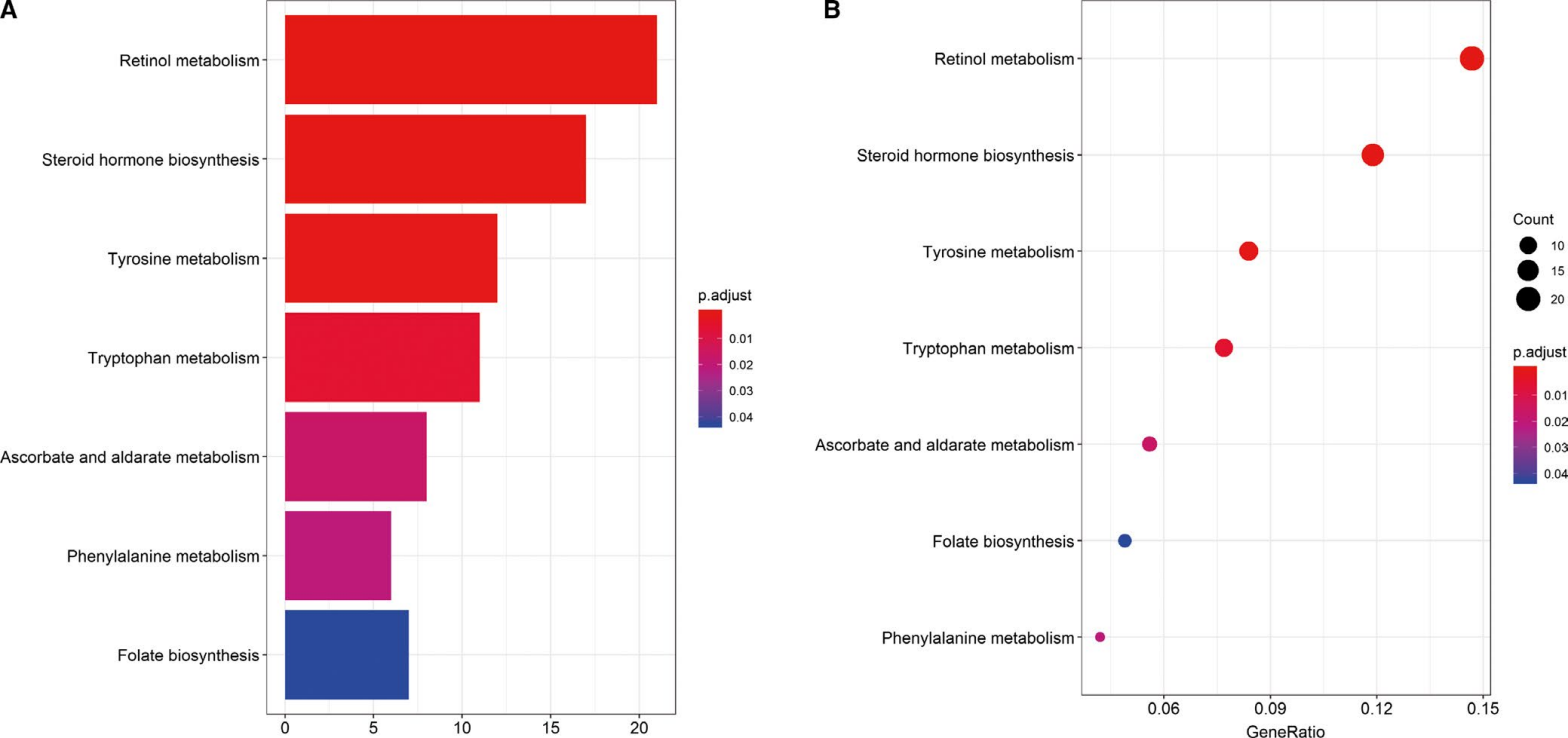
(A‐B) Kyoto Encyclopedia of Genes and Genomes (KEGG) pathway enrichment of differentially expressed mRNAs (DEMs) (*P* < 0.05)

The KEGG pathway analysis elucidated the potential biological functions, which were applied using the Iranges package (*P* < 0.05). The results showed that the DEMs were enriched in retinol metabolism, steroid hormone biosynthesis, and tyrosine metabolism (Figure 4).

### 3.3 Target prediction and construction of a ceRNA regulatory network in breast cancer

**Figure 5:**
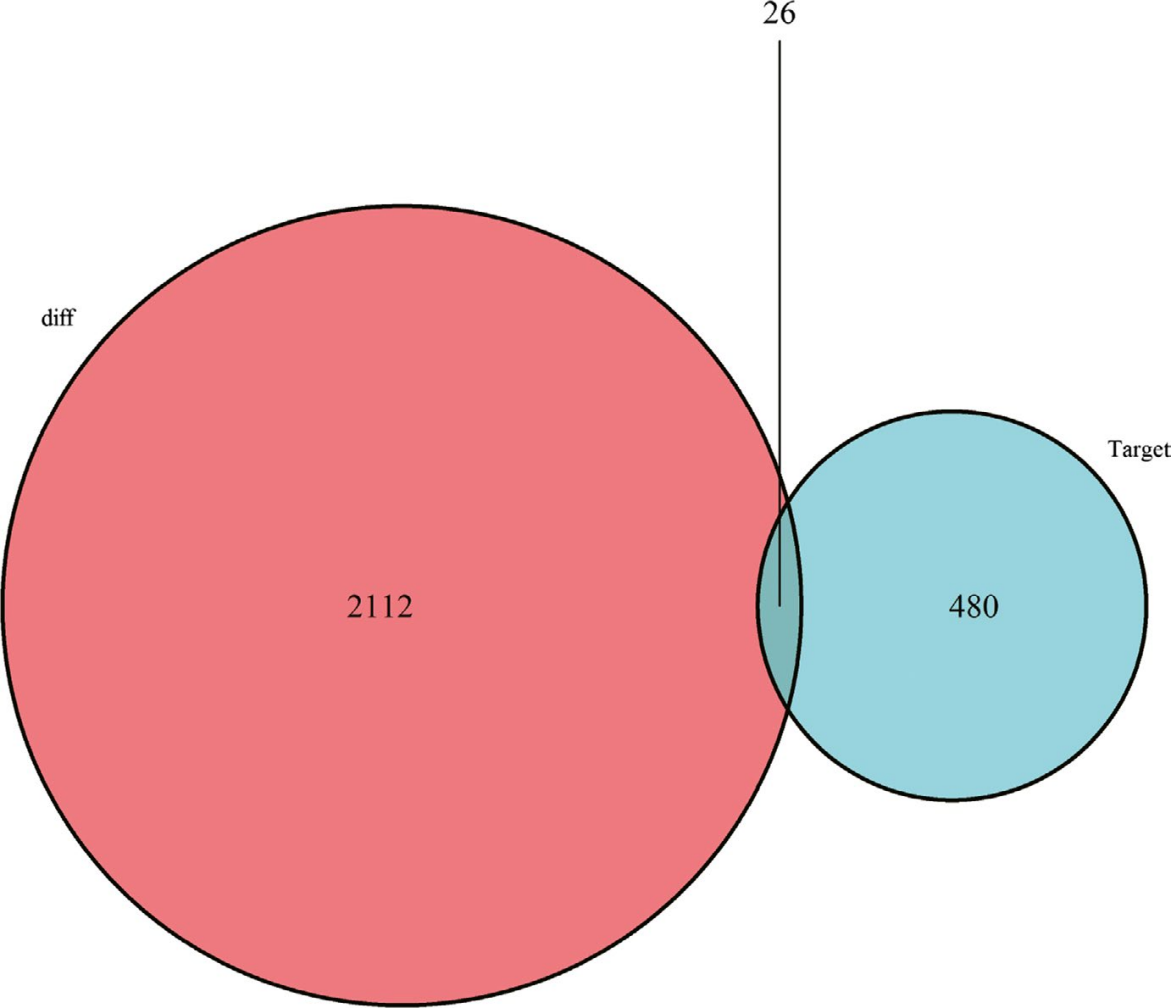
Venn diagram of differentially expressed mRNAs (DEMs) involved in the competing endogenous RNA (ceRNA) regulation network. The mRNAs expressed in the red area are all the DEMs in breast cancer. The target number is in the blue area, and the purple area represents the DEMs that are located in both the differential expression and target groups

Based on the Mircode database, we compared the DELs and DEMis, and the results revealed 323 DEMis‐DELs interactions, with 18 DEMis targeting 90 DELs (Table 4). We next conducted target prediction for 18 modified DEMis in all three databases (miRTarBase, miRDB, and TargetScan) and obtained 506 target genes (Table S4), which intersected with 2 138 DEMs, resulting in 26 DEMs (Figure 5). We then found that 26 DEMs were targeted by 12 DEMis (Table 5). To detect the significant relationships between the three intuitively, we constructed the ceRNA network using Cytoscape, which included 26 DEMs, 90 DELs, and 18 DEMis (Figure S1). Breast cancer is divided into four subtypes using the TCGA database: Ductal and Lobular Neoplasms, Cystic, Mucinous and Serous Neoplasms, Complex Epithelial Neoplasms, and other subtypes. Different breast cancer subtypes have different strategies for treatment, therefore the ceRNA networks of different subtypes were performed by using the same methods (Figures [Link], [Link], [Link], [Link]).

**Table 4:** 90 DELs targeted by 18 DEMis

DEMis	DELs
hsa‐mir‐141	AGAP11 LINC00518 AL513123.1 LINC00466 LINC00355 LINC00404 PLX6‐AS1 DSCAM‐AS1 LINC00484 AC009121.1 PDZRN3‐AS1 ADAMTS9‐AS2 HOTTIP PEX5L‐AS1 LINC00461
hsa‐mir‐200a	AGAP11 LINC00518 AL513123.1 LINC00466 LINC00355 LINC00404 PLX6‐AS1 DSCAM‐AS1 LINC00484 AC009121.1 PDZRN3‐AS1 ADAMTS9‐AS2 HOTTIP PEX5L‐AS1 LINC00461 AL589642.1
hsa‐mir‐145	AGAP11 SHANK2‐AS3 MIR7‐3HG LINC00518 DSCR4 TCL6 AF241725.1 LINC00243 SMCR2 PHEX‐AS1 LINC00337 LINC00113 ADIPOQ‐AS1 FAM155A‐IT1 SRGAP3‐AS2 ATXN8OS DLX6‐AS1 NDP‐AS1 ADAMTS9‐AS1 ADAMTS9‐AS2 LINC00461 ALDH1L1‐AS2 LINC00491 AL589642.1 LINC00052 LINC00261 PWPN1
hsa‐mir‐182	AGAP11 C15orf54 AC127496.1 LINC00221 TCL6 UCA1 LINC00243 PHEX‐AS1 LINC00337 ADIPOQ‐AS1 EMX2OS TLR8‐AS1 RBMS3‐AS3 MME‐AS1 ADAMTS9‐AS1 SYNPR‐AS1 ADAMTS9‐AS2 LINC00536 AL589642.1 AC040173.1 LINC00261
hsa‐mir‐206	AGAP11 C150rf54 LINC00518 TCL6 RMRP UCA1 LINC00488 LINC00243 AL356310.1 LINC00466 NAALADL2‐AS2 HOTAIR SRGAP3‐AS2 DLX6‐AS1 LINC00210 TLR8‐AS1 LINC00460 LINC00484 NDP‐AS1 CLRN1‐AS1 HOTTIP AC110619.1 LINC00261
hsa‐mir‐204	AGAP11 C2orf48 SHANK2‐AS3 AC127496.1 MIR7‐3HG LINC00305 C10orf91 LINC00518 LINC00221 TCL6 C1orf137 SMCR2 PHEX‐AS1 LINC00466 HOTAIR LINC00200 ATXN8OS DLX6‐AS1 LINC00210 TLR8‐AS1 DSCAM‐AS1 RBMS3‐AS3 CLRN1‐AS1 ADAMTS9‐AS2 HOTTIP AC061992.1 LINC00461 MAST4‐IT1 LINC00536 LINC00491 AL589642.1 LINC00524 LINC00261
hsa‐mir‐21	AGAP11 AC127496.1 DSCR4 LINC00221 TCL6 LINC00488 LINC00466 LINC00351 HOTAIR EMX2OS ATXN8OS ARHGEF7‐AS2 ADAMTS9‐AS1 AL139002.1 LINC00461 ALDH1L1‐AS2 AL589642.1 PWRN1
hsa‐mir‐375	AGAP11 C150rf54 LINC00518 TCL6 LINC00243 LINC00337 LINC00351 ADIPOQ‐AS1 HOTAIR ATXN8OS POU6F2‐AS2 ARHGEF7‐AS2 LINC00445 LSAMP‐AS1 SYNPR‐AS1 ADAMTS9‐AS2 OPCML‐IT1 C8orf49 LINC00261
hsa‐mir‐183	C2orf48 AC127496.1 TCL6 AC009093.1 AC513123.1 SMCR2 LINC00466 CHC1‐AS2 ADIPOQ‐AS1 NAALADL2‐AS2 LINC00392 LINC00200 EMX2OS ATXN8OS MYCNOS LSAMP‐AS1 ADAMTS9‐AS2 AC007731.1 AL589642.1 AC040173.1 LINC00261
hsa‐mir‐122	C2orf48 SHANK2‐AS3 AC127496.1 C10orf91 TCL6 AC009093.1 DSCR8 AC135178.1 RMRP TDRG1 UCA1 LINC00488 LINC00243 LGALS8‐AS1 PHEX‐AS1 ADIPOQ‐AS1 FAM155A‐IT1 LINC00355 ATXN8OS DLX6‐AS1 MYCNOS DSCAM‐AS1 LINC00484 ARHGEF‐AS2 NDP‐AS1 AC080129.1 ADAMTS9‐AS2 AC061992.1 LINC00461 LINC00491 C8orf49 AC110619.1 AL589642.1 PWPN1
hsa‐mir96	SHANK‐AS3 AC127496.1 LINC00221 TCL6 UCA1 LINC00488 LINC00243 PHEX‐AS1 LINC00466 LINC00210 RBMS3‐AS3 ADAMTS9‐AS1 SYNPR‐AS1 ADAMTS9‐AS2 LINC00461 LINC00536 AC040173.1
hsa‐mir‐187	SHANK2‐AS3 TCL6 SACS‐AS1 TLR8‐AS1 POU6F2‐AS2 CHL1‐AS1 LINC00484 ARHGEF7‐AS2 HOTTIP LINC00052
hsa‐mir‐301b	C150rf54 DSCR4 LINC00221 TCL6 PHEX‐AS1 HOTAIR ADAMTS9‐AS1 ADAMTS9‐AS2 AL139002.1 HOTTIP AC061992.1 ALDH1L1‐AS2 C8orf49 LINC00261
hsa‐mir‐429	C150rf54 C10orf91 AL356479.1 AC080037.1 LINC00466 DLX6‐AS1 LINC00460 CLRN1‐AS1 MME‐AS1 LINC00491 C8orf49 AL589642.1 AC040173.1 LINC00261
hsa‐mir‐210	AC127496.1 TCL6EMX2OS ATXN8OS ARHGEF‐AS2 AL139002.1 LINC00461 ALDH1L1‐AS2 AL589642.1
hsa‐mir‐144	LINC00305 DSCR4 TCL6 AL391421.1 LINC00488 LINC00466 ADIPOQ‐AS1 SACS‐AS1 DLX6‐AS1 FNDC1‐IT1 ADAMTS9‐AS1 ADAMTS9‐AS2 LINC00461 AC040173.1 PWRN1 LINC00261
hsa‐mir‐137	TCL‐6 DSCR8 AL391421.1 LINC00466 POU6F2‐AS2 CHL1‐AS1 DSCAM‐AS1 CLRN1‐AS1 ADAMTS9‐AS2 HOTTIP LINC00461 LINC00536 PWPN1
hsa‐mir‐184	UCA1 ADIPOQ‐AS1 EMX2OS BOK‐AS1 ADAMTS9‐AS2 HOTTIP LINC00491 OPCML‐IT1 C8orf49 AC110619.1 PWPN1

DELs, differentially expressed lncRNAs; DEMis, differentially expressed miRNAs.

**Table 5:** 26 DELs targeted by 12 DEMis

DEMis	DEMs
hsa‐mir‐204	TGFBR2 CHRDL1 KLHL40 CDH2 CREB5 HCAR2 SAMD5 NTRK2
hsa‐mir‐210	SERTM1
hsa‐mir‐21	SPRY2 CCL20
hsa‐mir‐183	AKAP12 CCNB1
hsa‐mir‐144	TTN FGF2 KPNA2
hsa‐mir‐200a	CCNE2
hsa‐mir‐182	CHL1 TCEAL7
hsa‐mir‐429	PARD6B SHCBP1 WASF3
hsa‐mir‐145	TGFBR2
hsa‐mir‐96	SLC1A1
hsa‐mir‐206	SFRP1
hsa‐mir‐137	KIT

DELs, differentially expressed lncRNAs; DEMs, differentially expressed mRNAs; DEMis, differentially expressed miRNAs.

### 3.4 Survival curve analysis of DELs, DEMs, and DEMis in the ceRNA network

**Figure 6:**
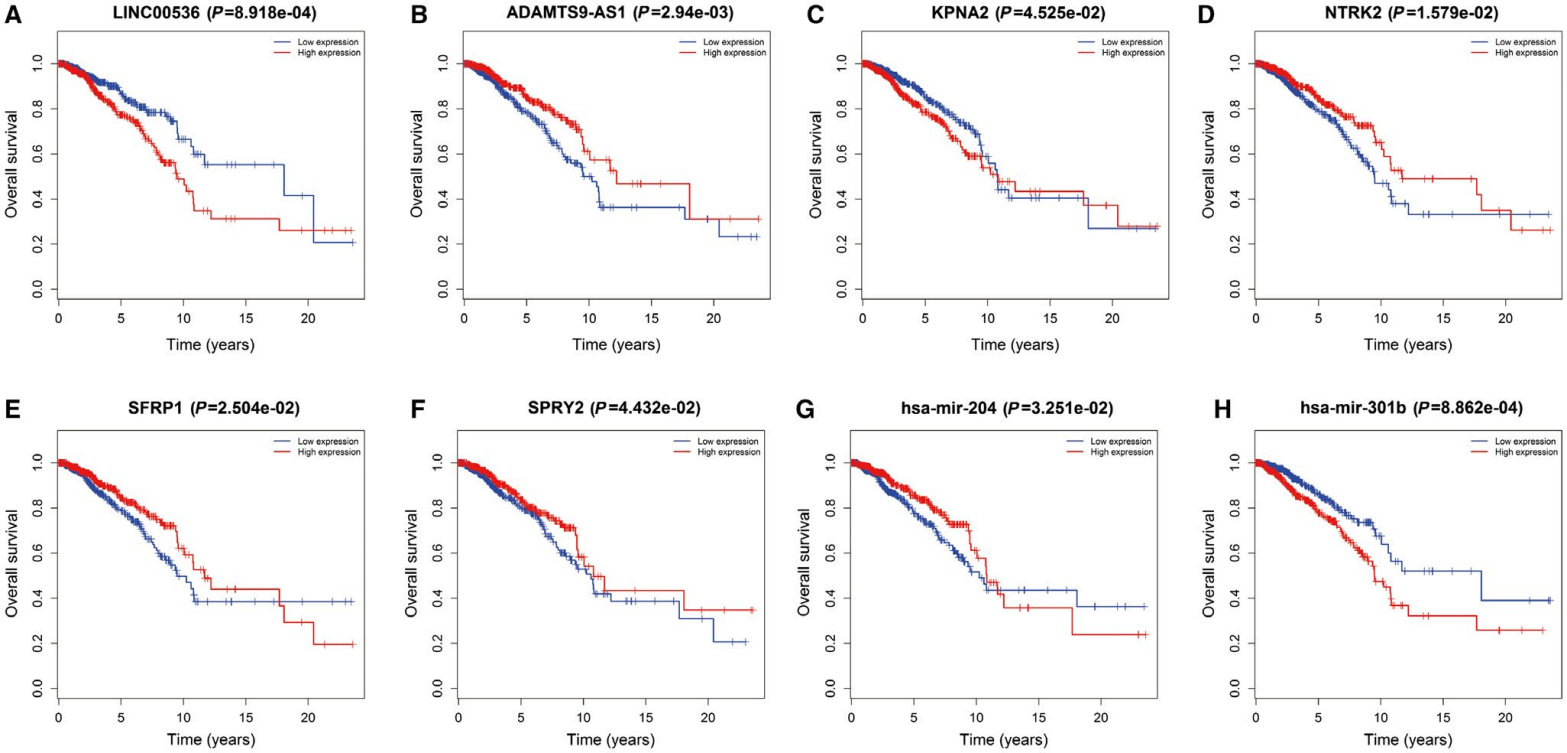
Survival curve analysis of differentially expressed lncRNAs (DELs), differentially expressed mRNAs (DEMs) and DEMis in the competing endogenous RNA (ceRNA) network

To explore the significance of the DELs, DEMs, and DEMis in the network, we investigated how their respective expression levels related to breast cancer patient survival using the Survival package of R (*P* < 0.05 was the threshold). The expression levels of LINC00536 (*P* = 8.1918e‐04) and ADAMTS9‐AS1 (2.94e‐03) were associated with poor OS, as well as KPNA2 (*P* = 4.525e‐02), NTRK2 (*P* = 1.579e‐02), SFRP1 (*P* = 2.504e‐02), SPRY2 (*P* = 4.432e‐02), mir‐204 (*P* = 3.251e‐02), and mir‐301b (*P* = 8.862e‐04) (Figure 6).

**Figure 7:**
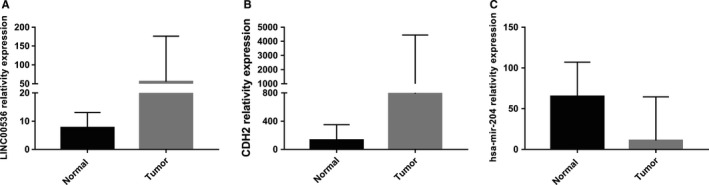
(A‐C) Expression of LINC00536, CDH2, and mir‐204 in normal and tumor tissues

**Figure 8:**
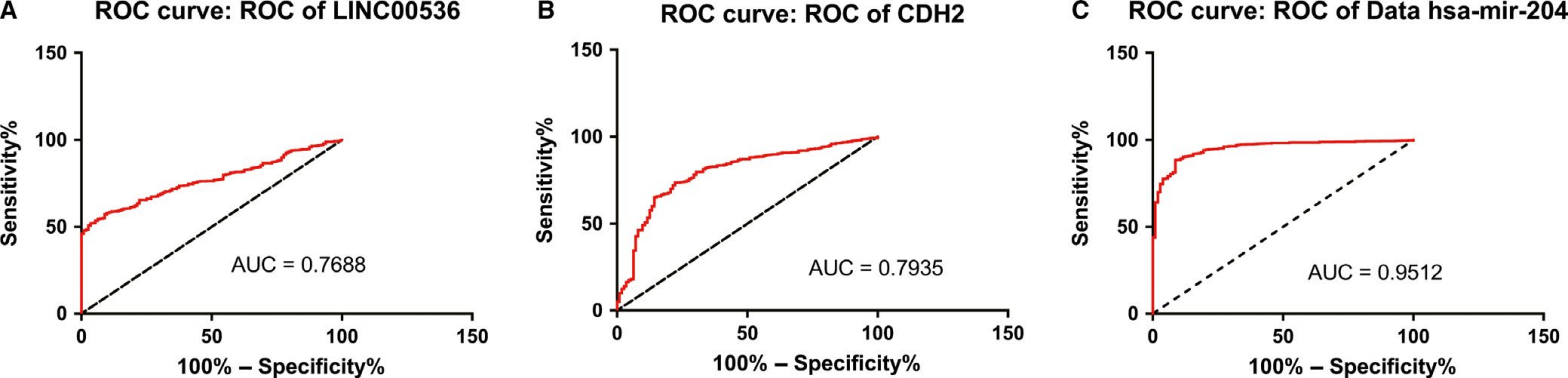
(A) ROC curve analysis for LINC00536 (AUC = 0.7688, 95% CI = 0.7365‐0.8011, *P* < 0.0001). (B) ROC curve analysis for CDH2 (AUC = 0.7935, 95% CI = 0.7507‐0.8363, *P* < 0.0001). (C) ROC curve analysis for mir‐204 (AUC = 0.9512, 95% CI = 0.9336‐0.9688, *P* < 0.0001)

Many studies have reported that mir‐204 is significant for breast cancer. From the network, we predicted that LINC00536 and CDH2 could combine with mir‐204 competitively from the ceRNA network. Using GraphPad Prism to analyze the expression levels of LINC00536, CDH2, and mir‐204, the results indicated that the expression levels of LINC00536 and CDH2 were increased in breast cancer (Figures 7A,B and 8A,B), while mir‐204 expression was decreased (Figures 7C and 8C). It can be speculated that the high expression of LINC00536 could bind to mir‐204, repressing the binding of CDH2 to mir‐204, thereby increasing the expression of CDH2. In addition, the LINC00536, CDH2, and mir‐204 expression levels in the GSE96670 dataset from GCBI website were consistent with the ceRNA network that we constructed.

### 3.5 Effects of LINC00536 expression on breast cancer cells

**Figure 9:**
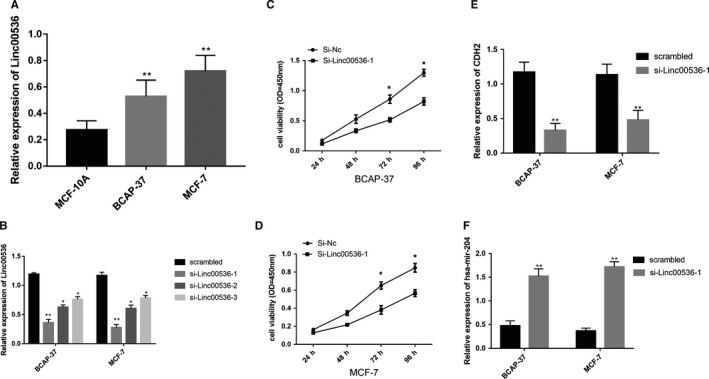
Effects of LINC00536 expression on CDH2 and mir‐204 in BC cells. (A) LINC00536 expression levels in MCF‐10A, MCF‐7, and BCAP‐37 cells were examined by quantitative real‐time PCR (qRT‐PCR). (B) LINC00536 expression levels after transfection with LINC00536‐siRNA in MCF‐7 and BCAP‐37 cells were determined by qRT‐PCR. (C and D) The cell proliferation levels were assessed using CCK‐8 in BCAP‐37 and MCF‐7 cells after transfection with LINC00536‐siRNA for 24, 48, 72, and 96 hours. (E and F) CDH2 and mir‐204 expression levels were evaluated by qRT‐PCR after transfection with LINC00536‐siRNA

To explore the function of LINC00536 in breast cancer cells, we performed qRT‐PCR in MCF‐7 and BCAP‐37 cells and in the normal breast cell line MCF‐10A. LINC00536 expression levels were upregulated in MCF‐7 and BCAP‐37 cells (Figure 9A). We then transfected LINC00536‐siRNA1, LINC00536‐siRNA2, and LINC00536‐siRNA3 into MCF‐7 and BCAP‐37 cells. LINC00536 expression was significantly decreased in LINC00536‐siRNA‐transfected cells compared with si‐NC (*P* < 0.001) (Figure 9B). LINC00536‐siRNA1 was selected for the subsequent assays. CCK‐8 proliferation assays suggested that suppression of LINC00536 in MCF‐7 and BCAP‐37 cells markedly promoted cell proliferation (Figure 9C,D). To confirm the interaction between LINC00536, CDH2, and mir‐204 in the ceRNA network in breast cancer cells, we detected the expression levels of mir‐204 and CDH2 in LINC00536‐siRNA1 breast cancer cells. The results indicated that after LINC00536‐siRNA1 treatment, CDH2 expression was reduced (Figure 9E), whereas mir‐204 expression was increased in transfected cells (Figure 9F). These findings were consistent with the ceRNA regulatory network that we constructed.

## 4 DISCUSSION

Although the 5‐year survival rate of breast cancer has improved in the past 10 years, breast cancer is still the main cause of cancer death in Chinese women.^20^ It is imperative to find more sensitive biomarkers and effective therapeutic targets.

In the ceRNA network constructed in this study, we found 90 DELs and 26 DEMs that were targeted by 18 common DEMis (Tables 4 and 6). Survival analysis of the genes in the network identified significant poor prognosis genes, including LINC00536, ADAMTS9‐AS1, KPNA2, NTRK2, SFRP1, SPRY2, mir‐204, and mir‐301b.

**Table 6:** Nucleotide sequences of primers used in the experiment

Gene name		Primer sequence
LINC00536 cDNA	Forward	5′‐CAGGACTACCGAGCACCAGGAC‐3′
	Reverse	5′‐TGACTCTCCTCAGCCAGCATCG‐3′
CDH2 cDNA Forward	Forward	5′‐AAGGTGGATGAAGATGGCATGGTG‐3′
	Reverse	5′‐TGCTGACTCCTTCACTGACTCCTC‐3′
Hsa‐mir‐204 cDNA Forward	Forward	5′‐CGCGTTCCCTTTGTCATCCT‐3′
	Reverse	5′‐AGTGCAGGGTCCGAGGTATT‐3′
LINC00536 siRNA1		5′‐GCGCTAAGGCAAATTGGATTGTGAA‐3′
LINC00536 siRNA2		5′‐GGGAGAAGATCAATATGCTAAACTT‐3′
LINC00536 siRNA3		5′‐CCTAGGAAGGGTAGTTTCATCAGAA‐3′

Many studies have reported that a low level of miR‐204 expression was associated with tumor progression in breast cancer.^21^, ^22^ Mir‐204 can directly target FOXA1 to regulate the invasion and metastasis of tumor cells^21^ and can affect tumor angiogenesis by regulating the expression of ANGPT1/TGFBR2.^22^ Chang et al suggested that miR‐130b‐5p from the miR‐301b‐130b cluster repressed the cyclin G2 gene in breast cancer cells.^25^ KPNA2 has been reported to regulate the subcellular localization of key proteins such as BRCA1, BARD1, PIAS1, RAD51, and CHK1, which are associated with poor prognosis.^26^ Howe et al proved that NTRK2 is directly targeted and downregulated by miR‐200c, thereby affecting the progression of EMT in breast cancer.^27^ It was reported that low expression of SFRP1 may activate the Wnt/β‐catenin signaling pathway to promote the proliferation, migration, and invasion of breast cancer cells.^28^ Overexpression of SPRY2 promoted EMT through upregulation of ZEB1 in colon cancer.^29^

In recent years, more attention has been paid to lncRNA research. In this study, we found several lncRNAs in the ceRNA network that were closely linked to breast cancer patients. Some of these lncRNAs have been shown to be associated with the development of breast cancer, such as HOTAIR, UCA1, and LINC00518,^30^, ^31^ but the research on the role of lncRNAs in breast cancer is still very scarce. From the survival analysis, LINC00536 and ADAMTS9‐AS1 were significantly associated with poor prognosis of breast cancer, but they have not been reported; therefore, they may become new therapeutic targets and prognostic biomarkers for breast cancer.

## CONFLICT OF INTEREST

The authors declare no competing interests.

## AUTHORS' CONTRIBUTIONS

Xiaojin Wang: Formal analysis, investigation, data curation, methodology, software, visualization, and writing‐original draft. Jiahui Wan: software, writing‐review, and editing. Zhanxiang Xu: Data curation and project administration. Shijun Jiang: Prepared all figures. Lin Ji: Data curation. Yutian Liu: Software and data curation. Shuwen Zhai: Editing. Rongjun Cui: Designed the experiments, and all authors reviewed the manuscript.
