# Supplementary figures and images for: Identification of competitive endogenous RNAs network in breast cancer
Source: Cancer Med. 2019 Apr 1;8(5):2392–403. doi: 10.1002/cam4.2099 (PMC6536941; doi:10.1002/cam4.2099)

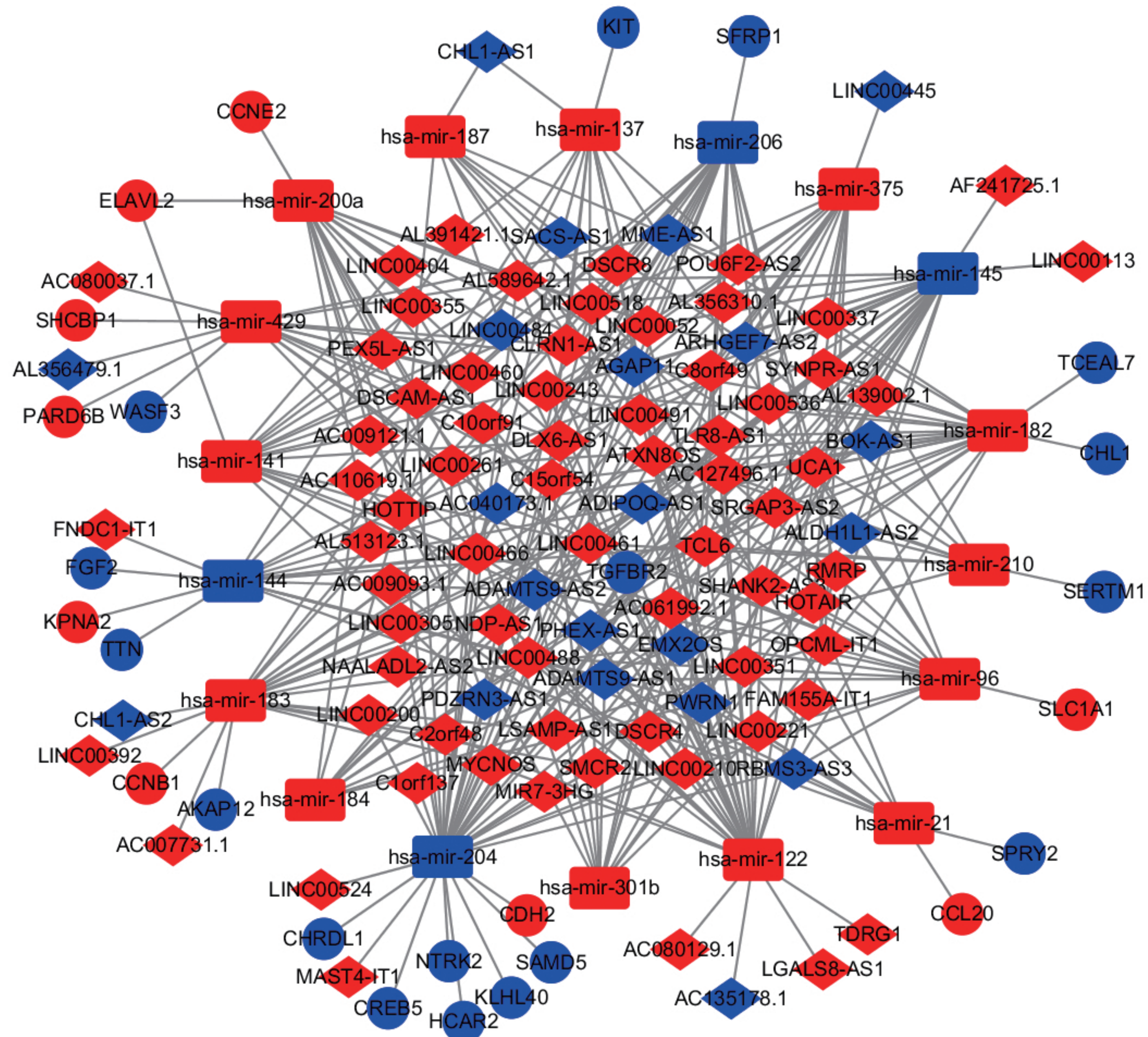

Supplement: Supplementary file 1 [file CAM4-8-2392-s001.pdf]

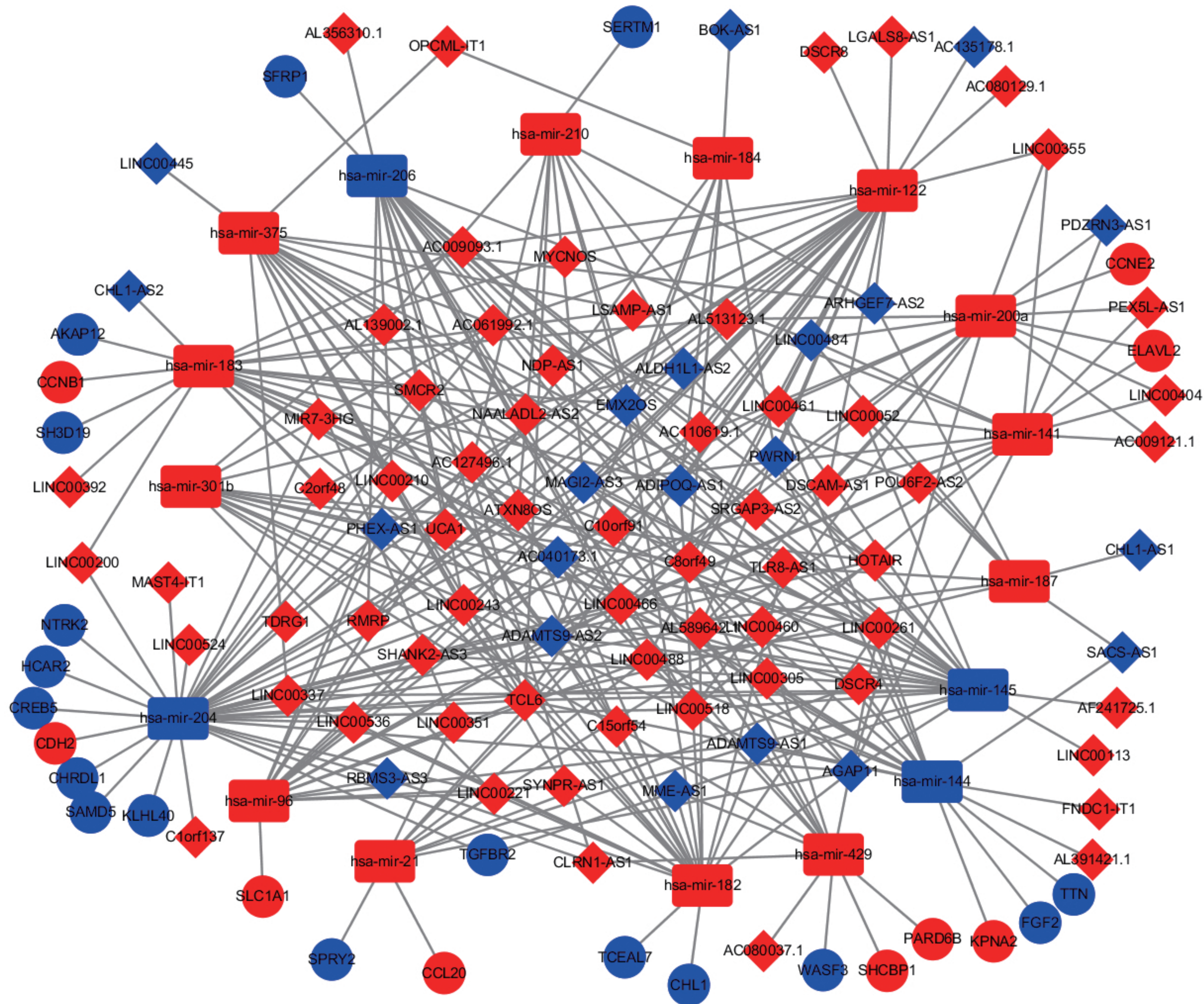

Supplement: Supplementary file 2 [file CAM4-8-2392-s002.pdf]

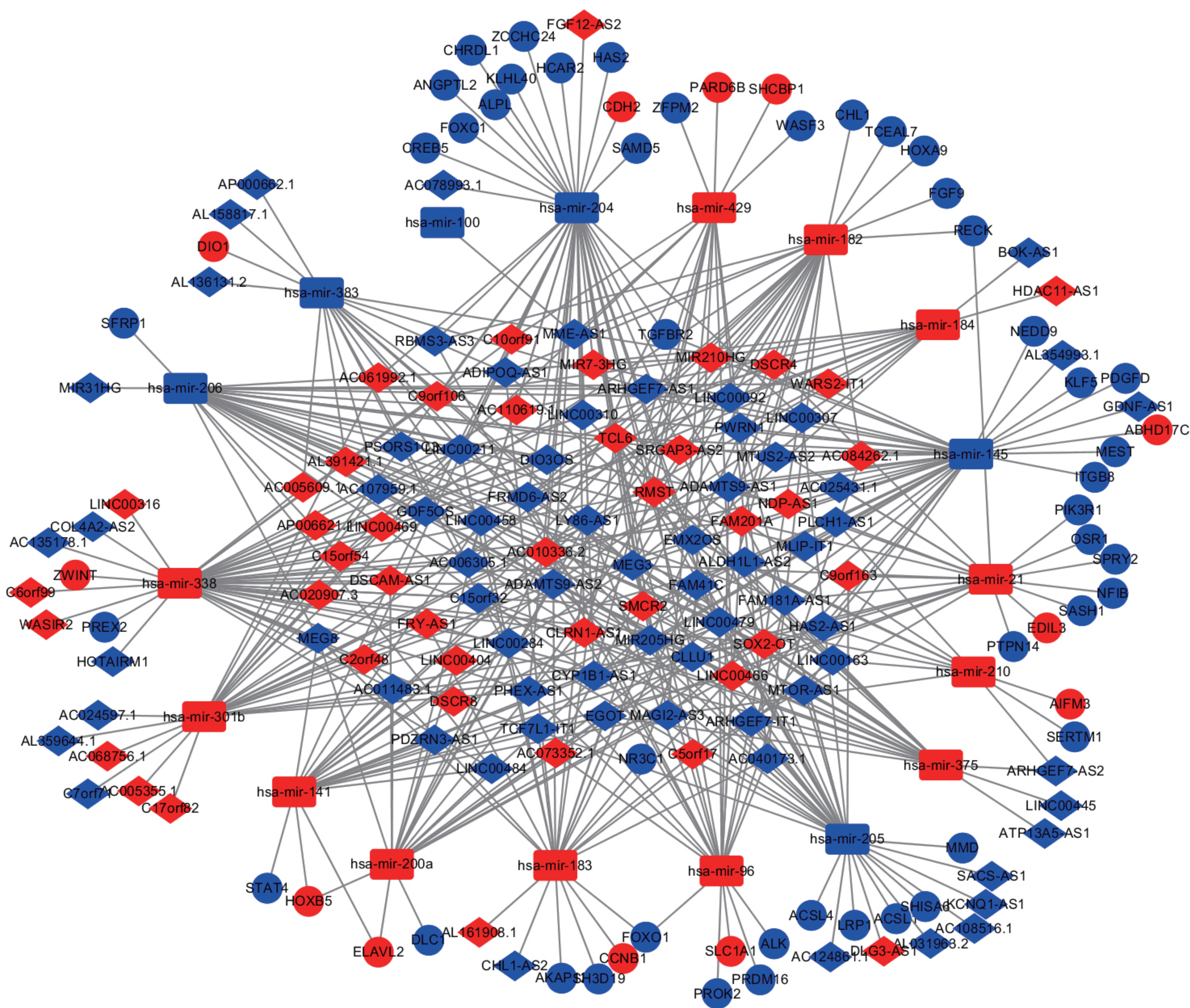

Supplement: Supplementary file 3 [file CAM4-8-2392-s003.pdf]

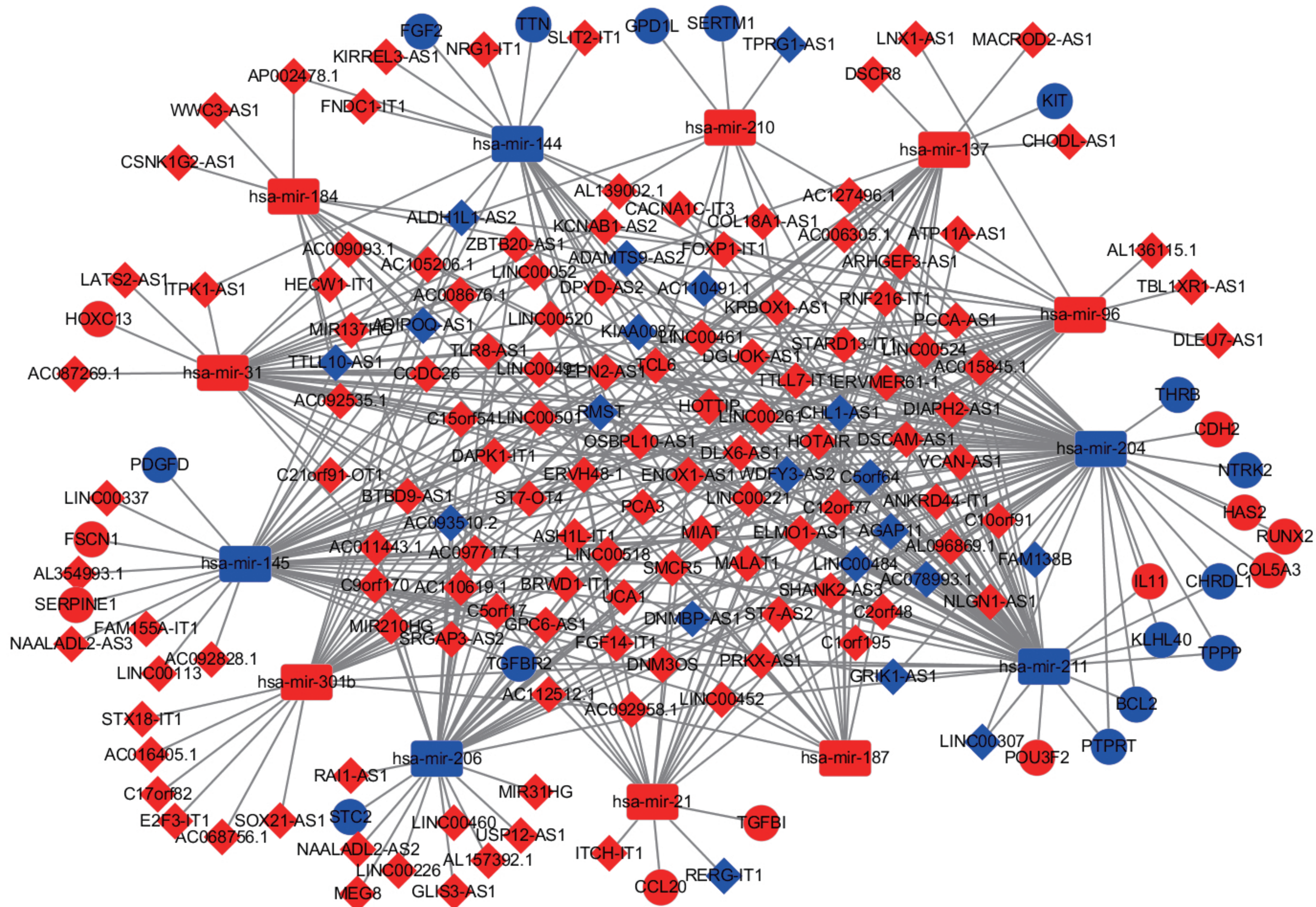

Supplement: Supplementary file 4 [file CAM4-8-2392-s004.pdf]

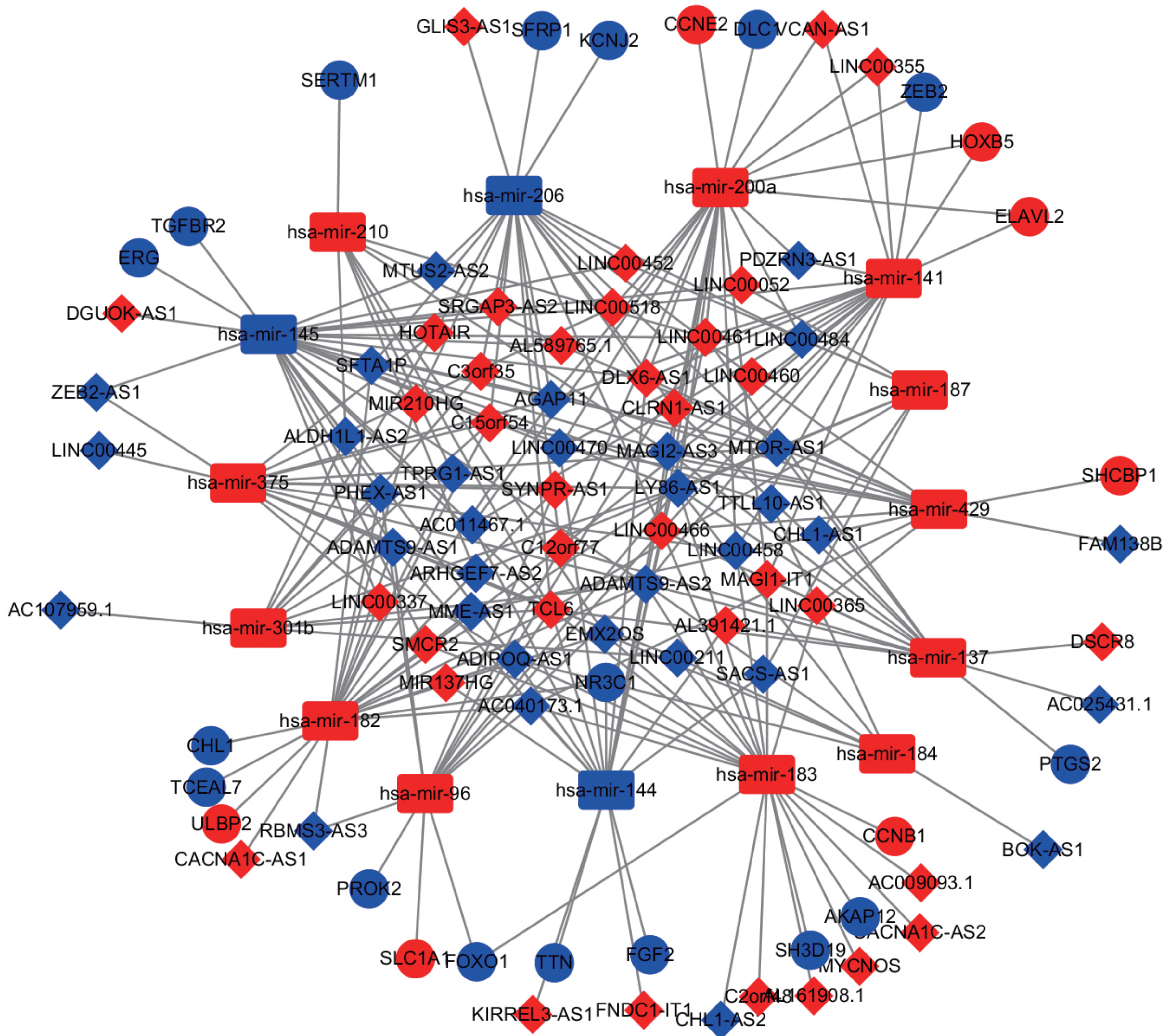

Supplement: Supplementary file 5 [file CAM4-8-2392-s005.pdf]
